# COVID-19 and medical education in Japan: a struggle for fairness and transparency

**DOI:** 10.5116/ijme.6512.8cb5

**Published:** 2023-10-06

**Authors:** Yudai Kaneda, Yuka Higuchi, Makoto Yoshida, Yoshika Saito

**Affiliations:** 1 School of Medicine, Hokkaido University, Hokkaido, Japan; 2 School of Medicine, Kyushu University, Fukuoka, Japan; 3 School of Medicine, Teikyo University, Tokyo, Japan; 4 School of Medicine, Kyoto University, Kyoto, Japan

**Keywords:** COVID-19, Medical Education, Fairness and Transparency, Institutional Reform, Japan

## To the Editor

The novel coronavirus disease 2019 (COVID-19) has profoundly impacted the learning environment of universities, especially medical schools, leading to a significant loss of educational opportunities. This situation has sparked a global controversy, with over 30% of medical students worldwide reporting anxiety, emphasizing the need for transformation in medical education.^1^ Indeed, reports indicate that approximately 5% of Japanese students who suspended their studies in the 2021 academic year did so due to the COVID-19 pandemic, emphasizing the need for a transformation in medical education that aligns with environmental changes and evolving student needs.^2^ In response to similar challenges, a medical school in Hungary, for example, provided study guidelines and COVID-19 stress management materials on demand, aiming to alleviate students' potential psychological anxiety about self-study during the pandemic.^3^ However, multiple reported instances in Japan suggest that the prioritization of learning opportunities for medical students was seemingly overlooked.

Illustrating the above challenges, a pre-medical student at the University of Tokyo (UT) was compelled to repeat a year due to absences resulting from a COVID-19 infection.^4^ The student contracted COVID-19 in May 2022 and could not attend classes or submit assignments, and although he had submitted a medical certificate, the assigned professor graded his assignment with zero points and did not allow him to undergo a make-up course because he missed the prescribed deadline. Although the student completed credits for other courses using alternative procedures, he was forced to repeat a year due to failing this one course that was not approved for such procedures. The student filed an appeal, but the response from the faculty was that the credit would not be granted even with a make-up course, and the course grade was inexplicably reduced by 17 points. Subsequently, the student held a press conference, demanding a justifiable explanation for the grade reduction and the absence of make-up courses. However, the UT faculty argued that since the student accessed their website on the evening of his absence, it was challenging to believe his COVID-19 symptoms were severe enough to prevent course participation. Additionally, they later revealed to the media that the point reduction was the faculty member's mistake in entering grades. As a result, the student deemed this response to be unfair and filed a lawsuit against the university requesting the implementation of make-up courses, which is still undergoing litigation without any resolution in sight.

Another case highlighting the disruptions in medical education can be seen at Gunma University, where an extraordinary circumstance arose. While approximately 90% of students in Japanese medical schools graduate within six years, 24 out of about 120 students at this university failed compulsory courses 'Medical Ethics' and 'Humanities in Medicine', taught by a specific professor.^5^ Moreover, this decision, where students were assessed based on impromptu performances simulating medical scenarios instead of test results aligned with the syllabus, sparked allegations of academic harassment from students and drew substantial media attention in October 2022. Indeed, this professor had a known history of academic harassment. Consequently, one of his students, who had taken a leave of absence after developing severe post-traumatic stress disorder as a result of such harassment, filed a lawsuit seeking damages from the university, yet the university continued to allow this professor to take classes despite the fact that this issue was unresolved. The response from the university to this issue was also inadequate, only involving the removal of the professor from teaching duties and offering make-up courses only to eight of the students who were in their third year. Consequently, such a series of insincere responses resulted in a decline in trust towards the university among the medical students, exacerbating their dissatisfaction and inciting controversy.

These cases highlight the potential neglect of students' needs by universities and faculties in Japan, drawing attention to the issue at a time when the global discourse centers on the loss of learning opportunities due to COVID-19. Amid the pandemic's chaos, faculty members, traditionally upholding the principle of infallibility common in Japanese organizations,^6^ may have been overwhelmed by the transition to new educational methods like online systems,^7^ which could have complicated their ability to prioritize and heed student voices. Nevertheless, failing to progress to the next academic year imposes a significant financial and mental burden on students in terms of tuition, living costs, and potential disadvantages to them regarding career progression, necessitating a swift and flexible response in both cases. Therefore, the bureaucratic approach of the faculties underscores the need for improved communication and a collective understanding in addressing such unprecedented situations.

Furthermore, a common problem highlighted by both cases was the need for more transparency in the processes of grade evaluation and implementation of make-up courses, indicating a wider systemic issue. Specifically, although the Japanese Ministry of Education, Culture, Sports, Science, and Technology instructed all universities to flexibly establish systems such as the provision of alternative learning if infected with COVID-19,^8^ it was suggested that merely issuing such directives by the government is insufficient in the actual context of medical education. To ensure objectivity and fairness in grade evaluations, universities must disclose the evaluation criteria to students in advance and assess them according to these criteria, promoting the achievement of educational objectives.^9^ Ambiguous grade evaluation criteria are contrary to these principles, and the failure to provide opportunities for re-examinations or make-up courses could be seen as an abuse of discretion, highlighting the need for a tra parent grade evaluation system.

In medical education, building adequate trust between faculty and students proves challenging when students' needs are overlooked, and the transparency of grade evaluations, along with the criteria for providing make-up courses, remains unclear. Therefore, involving students in the decision-making process is believed to be paramount to realizing a student-centric education. In fact, there is a growing global trend, prevalent in countries like the United States, to involve students directly in curriculum design, with several medical institutions empowering students to spearhead curriculum changes.^10^ In addition, by leveraging the shift towards online medical education due to COVID-19,^7^ mutual sharing and feedback on the realities of medical education with other universities through online platforms could effectively prevent similar incidents at specific institutions. Expanding such initiatives, continually incorporating student feedback into medical education, and constantly adapting the curriculum to meet the demands of the times could be potential solutions.

In conclusion, the COVID-19 pandemic has exposed significant deficiencies within the Japanese medical education framework. These shortcomings, particularly concerning the provision of make-up courses, transparency in grade evaluations, and faculty-student relationships, point to a need for systemic reforms and international cooperation. The visibility of such cases is typically low, and it remains unclear to what extent medical students worldwide are confronted with such circumstances. Consequently, these cases should not be trivialized as a problem in specific medical schools in Japan. These issues warrant a more comprehensive discussion and concerted efforts to establish an international consensus, aiming to foster a more flexible and adaptable environment for medical education, during both pandemics and normal circumstances.

### Conflicts of Interest

The authors declare that they have no conflict of interest.
